# The occurrence of germline *BRCA1 *and *BRCA2 *sequence alterations in Slovenian population

**DOI:** 10.1186/1471-2350-12-9

**Published:** 2011-01-14

**Authors:** Vida Stegel, Mateja Krajc, Janez Žgajnar, Erik Teugels, Jacques De Grève, Marko Hočevar, Srdjan Novaković

**Affiliations:** 1 Department of Molecular Diagnostics, Institute of Oncology Ljubljana, Zaloska 2, 1000 Ljubljana, Slovenia; 2 Unit of Genetic Counseling, Institute of Oncology Ljubljana, Zaloska 2, 1000 Ljubljana, Slovenia; 3 Department of Surgical Oncology, Institute of Oncology Ljubljana, Zaloska 2, 1000 Ljubljana, Slovenia; 4 Laboratory of Molecular Oncology, Oncologisch Centrum UZ Brussel, Laarbeeklaan 101, 1090 Brussels, Belgium

## Abstract

**Background:**

The *BRCA1 *and *BRCA2 *mutation spectrum and mutation detection rates according to different family histories were investigated in 521 subjects from 322 unrelated Slovenian cancer families with breast and/or ovarian cancer.

**Methods:**

The *BRCA1 *and *BRCA2 *genes were screened using DGGE, PTT, HRM, MLPA and direct sequencing.

**Results:**

Eighteen different mutations were found in *BRCA1 *and 13 in *BRCA2 *gene. Mutations in one or other gene were found in 96 unrelated families. The mutation detection rates were the highest in the families with at least one breast and at least one ovarian cancer - 42% for *BRCA1 *and 8% for *BRCA2*. The mutation detection rate observed in the families with at least two breast cancers with disease onset before the age of 50 years and no ovarian cancer was 23% for *BRCA1 *and 13% for *BRCA2*. The mutation detection rate in the families with at least two breast cancers and only one with the disease onset before the age of 50 years was 11% for *BRCA1 *and 8% for *BRCA2*. In the families with at least two breast cancers, all of them with disease onset over the age of 50 years, the detection rate was 5% for *BRCA2 *and 0% for *BRCA1*.

**Conclusion:**

Among the mutations detected in Slovenian population, 5 mutations in *BRCA1 *and 4 mutations in *BRCA2 *have not been described in other populations until now. The most frequent mutations in our population were c.181T > G, c.1687C > T, c.5266dupC and c.844_850dupTCATTAC in *BRCA1 *gene and c.7806-2A > G, c.5291C > G and c.3978insTGCT in *BRCA2 *gene (detected in 69% of *BRCA1 *and *BRCA2 *positive families).

## Background

Breast cancer represents 20.7% of all malignancies in females and is the most frequent type of cancer in the Slovenian female population. In 2007, 1147 new breast cancer cases were registered in Slovenia. The incidence rate among Slovenian women was 112/100000 and is slowly increasing. Breast and ovarian cancers together represent as much as 23.9% of all cancers in Slovenian females [1]. The majority of these two types of cancer are sporadic and only a small proportion (5%-10%) is known to be caused by dominantly inherited susceptibility genes [2]. The *BRCA1 *and *BRCA2 *genes were found to be mutated in a large number of families with multiple cases of an early onset of breast and ovarian cancer [3-6]. Mutations in *BRCA1 *and *BRCA2 *are highly penetrant and confer an increased risk of breast and ovarian cancer in carriers [7-9]. The cumulative breast cancer risk for *BRCA1 *and *BRCA2 *mutation carriers at the age of 70 was, in different studies, reported to be between 36% and up to 71% [10,11]. The studies of the Breast Cancer Linkage consortium based on the families with four or more cancer cases reported an approximate breast cancer risk of 80% at the age of 70 for mutation carriers [7,8,12]. The reported cumulative risk for ovarian cancer in *BRCA1 *and *BRCA2 *carriers at the age of 70 was 39% to 60% and 22% to 27%, respectively [8,13,14].

Up to now, several comprehensive studies performed throughout Europe determined the mutation profiles of *BRCA1 *and *BRCA2 *in families with history of breast and ovarian cancer. One of the largest studies carried out by a German consortium included 989 unrelated families with a history of breast and ovarian cancer who were screened for mutations in *BRCA1 *and *BRCA2*. The frequency of detected mutations in this study varied from 14% to 50% according to different family histories [15]. Another large study included 1010 unrelated families from the Czech Republic with an overall mutation detection rate of 29% for *BRCA1 *and *BRCA2 *[16]. A similar study was performed in the Dutch population which included 517 unrelated families, where an overall mutation detection of 23% was reported [17]. In French, Swedish and Finnish populations, 100 to 200 families were screened for *BRCA1 *or *BRCA2 *mutations [18-20]. The mutation detection rates in these studies varied from 10 to 25%. A higher mutation detection rate of 33% was observed in the study which included 42 Belgian families [21,22]. A comparably high overall mutation detection rate of 30% was reported also among 102 Spanish families [23]. In the Austrian population, the mutation detection rate was reported to be 20% for *BRCA1 *among 86 breast and ovarian cancer families [24]. In Italy, several reports were published. The overall mutation detection rate for *BRCA1 *and *BRCA2 *in these reports varied from 8 to 37% [25-29].

Slovenia is a country located in the central European area with approximately 2 million inhabitants. Since 1999, genetic counseling and testing for hereditary breast and ovarian cancer have been offered at the Institute of Oncology Ljubljana. Until now, approximately 1100 individuals have attended genetic counseling and 521 individuals from 322 families opted for genetic testing.

With this article, we wanted to present the *BRCA1 *and *BRCA2 *mutation spectrum and mutation detection rates according to different family histories in Slovenian the population.

## Methods

### Tested individuals

521 persons from 322 Slovenian families with breast and/or ovarian cancer underwent genetic testing. When possible, the proband tested for the presence of mutation in *BRCA1 *and *BRCA2 *genes in the family was the youngest individual affected by breast or ovarian cancer. All tested individuals provided written informed consent and attended genetic counseling sessions before and after testing.

All tested individuals were classified according to their family history. The family history data were verified in the Slovenian state cancer registry established in 1950.

The enrolled families were divided into 6 different groups according to the following criteria:

*Group A*: Families with two or more cases of breast cancer among first-degree relatives, including at least two cases with the disease onset under the age of 50, with no ovarian cancer.

*Group B*: Families with one or more cases of breast and at least one ovarian cancer among first-degree relatives.

*Subgroup BA *- families with at least one ovarian cancer and at least two breast cancers under the age of 50;

*Subgroup BC *- families with at least one ovarian cancer and at least two breast cancers, one under the age of 50;

*Subgroup BD *- Families with at least one ovarian cancer and at least two breast cancers, all beyond the age of 50;

*Subgroup 1B+1O *- Families with only one ovarian cancer and one breast cancer in the family or only one affected individual in the family, but having both types of cancer at the same time;

*Subgroup more O+1B *- Families with at least two ovarian cancers and only one breast cancer;

*Subgroup only O *- Families with at least two ovarian cancers and no breast cancer;

*Group C*: Families with two or more cases of breast cancer among first-degree relatives, including one case diagnosed before the age of 50 and no ovarian cancer.

*Group D*: Families with two or more cases of breast cancer among first-degree relatives, all diagnosed beyond the age of 50 and no ovarian cancer.

*Group E*: Families with a single case of female breast cancer diagnosed under the age of 40.

*Group MBR*: Families with a single breast cancer among first-degree relatives being the male breast cancer.

Bilateral breast cancers were counted as two independent cases of breast cancer.

*Controls: *The control group consisted of healthy volunteers, females aged from 50 to 69 years without any familial and personal history of breast and/or ovarian cancer who were tested for the presence of all mutations, unclassified variants (UV) and polymorphisms found in patients.

### Mutation screening

The DNA was isolated from peripheral blood using the DNA blood isolation kit (Qiagen, Hilden, Germany).

Mutation screening in *BRCA1 *and *BRCA2 *genes was performed by two laboratories - the Laboratory of Molecular Oncology at the Vrije University Brussels, Belgium and the Laboratory of Molecular Diagnostics at the Institute of Oncology Ljubljana, Slovenia.

All samples were, at the beginning, tested for six most frequent mutations in the Slovenian population (c.7806-2A > G in *BRCA2*, c.5266dupC, c.1687C > T, c.191G > A, c.181T > G, c.181T > A in *BRCA1*) [30,31].

In the Laboratory of the Vrije University Brussels, screening for six most frequent mutations was done by denaturing gradient gel electrophoresis (DGGE) for mutations c.5266dupC, c.191G >A, c.181T > G, c.181T > A in *BRCA1 *and c.7806-2A > G in *BRCA2 *and by protein truncation test (PTT) for mutation c.1687C > T in *BRCA1*. The additional screening of *BRCA1/2 *was performed with PTT (screening of exon 11 in *BRCA1 *and exons 10 and 11 in *BRCA2*) and DGGE (the rest of exons of *BRCA1 *and *BRCA2*) [21].

In the Laboratory of the Institute of Oncology Ljubljana, screening for six most frequent mutations was done using fluorescent hybridization probes on Light Cycler 2.1 for c.5266dupC, c.1687C > T, c.191G > A, c.181T > G in *BRCA1*, high-resolution melting - HRM on Light Cycler 480 II (Roche Molecular Biochemicals, Mannheim, Germany) for c.181T > A in *BRCA1 *and restriction length polymorphism for c.7806-2A > G in *BRCA2 *[30,31]. The additional screening of *BRCA1/2 *was performed with DGGE.

In both laboratories, the DGGE was performed using primers designed by Ingeny and according to the manufacturer's instructions (Ingeny International BV, Goes, Netherlands).

Samples presenting migration abnormalities were further sequenced on the automated sequence analyzer ABI310 (Applied Biosystems, USA) (using the BDT v.1.1 and performing the labeling PCR reaction) according to manufacturer's instructions. The samples were screened for the presence of large deletions and duplications using the multiplex ligation-dependent probe amplification MLPA kit (MRC Holland, Amsterdam, Netherlands). For *BRCA1 *gene, the MLPA analysis was performed with probe set P002 and confirmed by probe set P087, and for *BRCA2 *with probe set P045-B1. The deletion breakpoints were not characterized at the sequence level.

The description of nucleotide sequence variations is in accordance with HGVS nomenclature, and in tables, also in accordance with BIC nomenclature [32,33]. According to HGVS nomenclature DNA variants are numerated according to NCBI reference sequence NM_007294.2 for *BRCA1*, NM_000059.3 for *BRCA2*. The first nucleotide of the start codon ATG is numerated as 1. According to BIC nomenclature, DNA variants are numerated according to NCBI reference sequence HSU14680 for mRNA of BRCA1, or U43746 for mRNA of BRCA2. The first nucleotide of mRNA is numerated as 1.

Statistical analysis was performed using the Chi-square test.

## Results

The study was performed on 521 individuals from 322 Slovenian breast and/or ovarian cancer families. In 96 families, either the mutation in *BRCA1 *or the mutation in *BRCA2 *was detected. The overall mutation detection rate for *BRCA1 *and *BRCA2 *genes was 29.8% (96/322). Thus, in 226 families, we found no pathogenic mutation in either of the two investigated genes.

### BRCA1 mutation spectrum

In *BRCA1*, 18 different deleterious mutations were found in 68 families (Table 1). The four most common mutations (c.181T > G, c.1687C > T, c.5266dupC and c.844_850dupTCATTAC) were detected in 44 families (65%). The additional 14 mutations in *BRCA1 *appeared in other 24 families.

**Table 1:** Mutations in *BRCA1 *gene in Slovenian population.

BIC nomenclature*	HGVS nomenclature**	Protein change according to BIC database	Described in BIC database (No. of quotations in BIC)	Present in other than Slovenian population (No. of families) ***	No. of positive Slovenian families
235G > A	c.116G > A	C39Y	yes (4)		3

300T > G	c.181T > G	C61G	yes (202)	Germany (30), Italy (?), Czech rep (20), Austria (3), Slovakia (2)	15

300T > A	c.181T > A	C61S	no		4

310G > A	c.191G > A	C64Y	yes (20)	Germany (1), Czech rep (1), Canada (2)	1

576delAG	c.457_458delAG	157X	no		1

963-969 dup (969ins7)	c.844_850dupTCATTAC	288X	yes (2)	Germany (2)	8

962 del4	c.843_846delCTCA	297X	yes (17)	Germany (2), Italy (?), Czech rep (1), Austria (2), Slovakia (2), Canada (2- Slavic origin)	2

1806C > T	c.1687C > T	Q563X	yes (88)	Germany (5), Czech rep (6), Austria (2)	13

2388delG	c.2269delG	764X	yes (7)		1

3137delTTCA	c.3018_3021delTTCA	1022X	yes (2)		1

5296 del4 (GAAA)	c.5177_5180delGAAA	1728X	yes (45)		1

5370C > T	c.5251C > T	R1751X	yes (30)	Germany (1),	1

5382insC	c.5266dupC	1829X	yes (200)	Germany (46), Italy (?), Czech rep. (75), Netherlands (5), Austria (2), Slovakia (2)	8

5496A > T	c.5377A > T	K1793X	no		2

Ex1-2del	Ex1-2del		no	Germany (?),Czech (1) Canada, Spain	1

Ex5-10 del	Ex5-10 del		no		3

Ex5-8 del	Ex5-8 del		no		1

Ex5-7 del	Ex5-7 del		no	Spain, Germany	2

Total BRCA1					68

Description of nucleotide variations is in accordance with *BIC nomenclature (DNA variants are numerated according to NCBI reference sequence HSU14680 for mRNA of *BRCA1*; the first nucleotide of mRNA is numerated as 1) or **HGVS nomenclature (DNA variants are numerated according to NCBI reference sequence NM_007294.2 for *BRCA1*; the first nucleotide of the start codon ATG is numerated as 1).

*** References: Spain [23,44]. Czech Rep [16,45]. Germany [15,46]. Italy [15]. Netherlands [17]. Austria [24]. Slovakia [47]. Canada [48].

Four of the mentioned 18 mutations are missense mutations affecting the 5'RING domain (c.116G > A, c.181T > G, c.181T > A, c.191G > A), three are nonsense mutations (c.1687C > T, c.5251C > T and c.5377A > T), seven are frame-shift mutations (c.457_458delAG, c.844_850dupTCATTAC, c.843_846delCTCA, c.2269delG, c.3018_3021delTTCA, c.5177_5180delGAAA and c.5266dupC) and four are deletions of the whole exons (Ex1-2 del, Ex5-7 del, Ex5-8 del and Ex5-10 del).

The most common mutation found in *BRCA1 *gene was c.181T > G. It was detected in 15 families. The patients with ovarian cancer represented 36% of all breast and ovarian cancer cases in the families bearing the mutation c.181T > G. The average age of the patients at the onset of ovarian cancer was 52.1 years and the average age of the patients at the onset of breast cancer was 48.1 years in these families, respectively. Breast cancer was, in these families, in 56% of cases detected before the age of 50. In five families, colorectal cancer, and in two families, cancers of the uterus, were also reported in the family history (46% of c.181T > G families have the HNPCC (hereditary non-polyposis colon cancer) related cancers in their family history).

The second most common mutation in *BRCA1 *gene in Slovenian families with breast and/or ovarian cancer was c.1687C > T. This mutation was observed in 13 families. The patients with ovarian cancer represented 32% of all breast and ovarian cancer cases in the families bearing the mutation c.1687C > T. In these families the average age of the patients at the onset of ovarian cancer was 52.5 years and the average age of the patients at the onset of breast cancer was 46.0. In these families, in 53% of cases, breast cancer was detected before the age of 50.

The mutation c.5266dupC in *BRCA1 *gene was detected in 8 families. The patients with ovarian cancer represented just 9% of all breast and ovarian cancer cases in the families bearing the c.5266dupC mutation since it was diagnosed in merely 2 cases (onset at 50 and 48 years, respectively). The average age of the patients at the onset of breast cancer in these families was 37.3 years. In more than 89% of cases, breast cancer was diagnosed before the age of 50.

The mutation c.844_850dupTCATTAC in *BRCA1 *gene was noticed in 8 families. The patients with ovarian cancer represented 30% of all breast and ovarian cancers cases in the families bearing the mutation c.844_850dupTCATTAC. The average age of the patients at the onset of ovarian cancer was 53.7 years, while the average age of the patients at the onset of breast cancer was 47.5 years. In 69% of cases, breast cancer was diagnosed before the age of 50.

### BRCA2 mutation spectrum

The analysis of the *BRCA2 *gene revealed 13 different mutations in 28 families (Table 2). The most common mutation in *BRCA2 *gene is a splice site mutation c.7806-2A > G. It was detected in 11 families (which represents 39% of all *BRCA2 *positive families). The remaining 12 *BRCA2 *mutations were much less frequent in the Slovenian population (each being discovered in 1 to 4 families) (Table 2). They are nonsense mutations or frame-shift mutations causing a truncation of the encoded protein. No large deletions were found in the *BRCA2 *gene.

**Table 2:** Mutations in *BRCA2 *gene in Slovenian population.

BIC nomenclature*	HGVS nomenclature**	Protein change according to BIC database	Described in BIC database (No. of quotations in BIC)	Present in other than Slovenian population (No. of families) ***	No. of positive Slovenian families
1003A > T****	c.775A > T	R259X	no		1

1756G > T	c.1528G > T	E510X	yes (1)		1

2041insA	c.1813dupA	615X	yes (97)	Germany (5), Czech rep (2)	1

3493C > T	c.3265C > T	Q1089X	yes (1)		1

4206 ins4 (TGCT) 4203_4206dupl	c.3978insTGCT c.3975_3978dupTGCT	1330X	yes		3

5164delGAAA	c.4936_4939delGAAA	1668X	yes		1

5519C > G	c.5291C > G	S1764X	no		4

5579insA	c.5351insA c.5351dupA	1786X	yes	Netherland (6)	1

5837TC > AG	c.5609_5610delTCinsAG	F1870X	yes (2)		1

6719delAGTT	c.6491_6494delAGTT	2166X	no		1

7531C > T	c.7303C > T	Q2435X	no		1

IVS16-2A > G 8064-2A > G	c.7806-2A > G	aberrant splicing	yes (5)	Germany (2 ), Italy (2)	11

9514C > T	c.9286C > T	E3096X	yes (1)	no	1

Total					28

Description of nucleotide variations is in accordance with BIC nomenclature* (DNA variants are numerated according to NCBI reference sequence U43746 for mRNA of *BRCA2*; the first nucleotide of mRNA is numerated as 1) or HGVS nomenclature** (DNA variants are numerated according to NCBI reference NM_000059.3 for *BRCA2*; the first nucleotide of the start codon ATG is numerated as 1). ***References: Germany [15]. Italy [15]. Czech Rep [16,45]. Netherland [17]. ****Albanian origin - Kosovo.

The c.7806-2A > G mutation was observed in 11 families. The patients with ovarian cancer (three patients aged 50, 59 and 70 years at the onset of disease) represented only 6% of all breast and ovarian cancer cases in the families bearing the mutation c.7806-2A > G. The average age of the patients at the onset of breast cancer was 50.9 years. Breast cancer was in 40% of cases discovered before the age of 50.

The second most frequent mutation in *BRCA2 *gene was c.5291C > G. It was detected in four families. The average age of the patients at the onset of breast cancer in these families was 45.5 years. In 72% of cases, breast cancer was discovered before the age of 50 years. No ovarian cancers were observed in these families.

The third most common mutation in *BRCA2 *gene occurring in three families was c.3975_3978dupTGCT. The mutation c.3975_3978dupTGCT is located in the "ovarian cluster region" of *BRCA2 *gene [34,35]. The patients with ovarian cancer (two patients aged 40 and 52 years at the onset of the disease) represented 29% of all breast and ovarian cancer cases in these families. The average age of the patients at the onset of breast cancer was 52.2 years. In 66% of cases, breast cancer was detected before the age of 50 years.

### Polymorphisms and unclassified sequence variants in BRCA1 and BRCA2 genes

All sequence variants which were not classified as mutations were categorized either as polymorphisms or as unclassified variants (UVs) according to the BIC database (Breast cancer information core) (Tables 3, 4 and 5). When the sequence variant was not reported in the BIC database, we classified it as a UV. Polymorphisms detected in individuals with breast and ovarian cancer family history and those found in the control group are listed in Table 3, while unclassified variants are given in Tables 4 and 5.

**Table 3:** Polymorphisms in *BRCA1 *and *BRCA2 *genes in Slovenian population.

BIC nomenclature*	HGVS nomenclature**	Protein change	Allele	No. of alleles/all alleles of tested probands (allele frequencies)	No. of alleles/all alleles of tested healthy individuals (allele frequencies)	Described in BIC database	Clinical importance entered in BIC database (allele frequencies)
**BRCA1**							

710 C > T	c.591C > T	C197C	T	1/380 (0.003)	0/80 (0.000)	yes	no (0.02)

1186A > G	c.1067A > G	Q356R	G	25/380 (0.065)	6/80 (0.075)	yes	no (0.06)

2196G > A	c.2077G > A	D693N	A	6/208 (0.029)	4/80 (0.050)	yes	no (0.08)

2201C > T	c.2082C > T	S694S	T	66/208 (0.32)	25/80 (0.31)	yes	no (0.31)

2430T > C	c.2311T > C	L771L	C	66/208 (0.32)	25/80 (0.31)	yes	no (0.31)

2731C > T	c.2612C > T	P871L	T	66/208 (0.32)	25/80 (0.31)	yes	no (0.34)

3232A > G	c.3113A > G	E1038G	G	66/208 (0.32)	25/80 (0.31)	yes	no (0.31)

3667A > G	c.3548A > G	K1183G	G	66/208 (0.32)	25/80 (0.31)	yes	no (0.31)

4427T > C	c.4308T > C	F1436S	C	66/208 (0.32)	25/80 (0.31)	yes	no (0.31)

4956A > G	c.4837A > G	S1613G	G	66/208 (0.32)	25/80 (0.31)	yes	no (0.31)

**BRCA2**							

203G > A	c.1-25G > A	5'-UTR	A	66/208 (0.32)	25/80 (0.31)	yes	no (0.25)

1093A > C	c.865A > C	N289H	C	18/380 (0.047)	3/80 (0.038)	yes	no (?)

1342C > A	c.1114C > A	H372N	A	160/208 (0.77)	56/80 (0.70)	yes	no (0.72)

1379C > T	c.1151C > T	S384F	T	1/208 (0.005)	0/80 (0.000)	yes	no (?)

1593A > G	c.1365A > G	S455S	G	7/208 (0.034)	3/80 (0.038)	yes	no (0.01)

2020A > G	c.1792A > G	T598A	G	4/208 (0.019)	0/80 (0.000)	yes	no (?)

2457T > C	c.2229T > C	H743H	C	18/380 (0.047)	5/80 (0.063)	yes	no (?)

3199A > G	c.2971A > G	N991D	G	7/208 (0.034)	3/80 (0.038)	yes	no (?)

3624A > G	c.3396A > G	L1132L	G	73/208 (0.35)	38/80 (0.48)	yes	no (0.31)

4035T > C	c.3807T > C	V1269V	C	27/208 (0.130)	18/80 (0.225)	yes	no (0.19)

4486G > T	c.4258G > T	D1420Y	T	3/208 (0.014)	0/80 (0.000)	yes	no (?)

5427C > T	c.5199C > T	S1733S	T	4/208 (0.019)	4/80 (0.050)	yes	no (?)

7470A > G	c.7242A > G	S2414S	G	57/208 (0.27)	21/80 (0.26)	yes	no (0.21)

8034-14C > T	c.7806-14C > T	intron	T	95/208 (0.46)	33/80 (0.41)	yes	no (0.50)

8410G > A	c.8182G > A	V2728I	A	1/208 (0.005)	1/80 (0.013)	yes	no (?)

10323delCins11	10095delCins11	3369X	del	3/380 (0.008)	0/80 (0.000)	yes	no (?)

Description of nucleotide variations is in accordance with BIC nomenclature* (DNA variants are numerated according to NCBI reference sequence HSU14680 for mRNA of *BRCA1*,or U43746 for mRNA of *BRCA2*; the first nucleotide of mRNA is numerated as 1) or HGVS nomenclature** (DNA variants are numerated according to NCBI reference sequence NM_007294.2 for *BRCA1*, NM_000059.3 for *BRCA2*; the first nucleotide of the start codon ATG is numerated as 1). UTR - untranslated region. (?)-data unknown.

**Table 4:** Unclassified sequence variants in *BRCA1 *gene in Slovenian population.

BIC nomenclature*	HGVS nomenclature**	Protein change	Allele	No. of alleles/all alleles of tested probands (allele frequencies)	No. of alleles/all alleles of tested healthy individuals (allele frequencies)	Described in BIC database	Clinical importance entered in BIC database
**Missense changes**							

212C > G	c.93C > G	I31M	G	3/380 (0.008)	0/80 (0.000)	yes	unknown

462C > A	c.343C > A	P115S	A	1/208 (0.005)	0/80 (0.000)	no	

1112G > C	c.993G > C	R331S	C	2/208 (0.010)	0/80 (0.000)	yes	unknown

3238G > A	c.3119G > A	S1040N	A	5/208 (0.024)	0/80 (0.000)	yes	unknown

3421G > A	c.3302G > A	S1101N	A	1/208 (0.005)	0/80 (0.000)	yes	unknown

3573G > A	c.3454G > A	D1152N	A	1/208 (0.005)	0/80 (0.000)	yes	unknown

3768T > C	c.3649T > C	S1217P	C	1/208 (0.005)	0/80 (0.000)	no	

4158A > G	c.4039A > G	R1347G	G	2/208 (0.010)	0/80 (0.000)	yes	unknown

4384G > A	c.4265G > A	G1422E	A	1/208 (0.005)	0/80 (0.000)	no	

4675A > G	c.4556A > G	N1519S	G	1/208 (0.005)	0/80 (0.000)	no	

5075G > A	c.4956G > A	M1652I	A	3/208 (0.014)	0/80 (0.000)	yes	unknown

5124G > T	c.5005G > T	A1669S	T	1/208 (0.005)	0/80 (0.000)	yes	unknown

Description of nucleotide variations is in accordance with BIC nomenclature* (DNA variants are numerated according to NCBI reference sequence HSU14680 for mRNA of *BRCA1*; the first nucleotide of mRNA is numerated as 1) or HGVS nomenclature** (DNA variants are numerated according to NCBI reference sequence NM_007294.2 for *BRCA1*; the first nucleotide of the start codon ATG is numerated as 1). UV- unclassified variant.

**Table 5:** Unclassified sequence variants in *BRCA2 *gene in Slovenian population.

BIC nomenclature*	HGVS nomenclature**	Protein change	Allele	No. of alleles/all alleles of tested probands (allele frequencies)	No. of alleles/all alleles of tested healthy individuals (allele frequencies)	Described in BIC database	Clinical importance entered in BIC database
**Missense changes**							

2032G > A	c.1804G > A	G602R	A	1/208 (0.005)	0/80 (0.000)	yes	unknown

3747C > T	c.3519C > T	S1152L	T	1/208 (0.005)	0/80 (0.000)	no	

5078G > A	c.4850G > A	S1617N	A	1/208 (0.005)	0/80 (0.000)	no	

5972C > T	c.5744C > T	T1915M	T	14/208 (0,067)	3/80 (0.038)	yes	unknown

8107A > T	c.7879A > T	I2627F	T	1/380 (0.003)	0/80 (0.000)	yes	unknown

8482A > T	c.8254A > T	I2752F	T	1/208 (0.005)	0/80 (0.000)	yes	unknown

8579G > A	c.8351G > A	R2784Q	A	2/380 (0.005)	0/80 (0.000)	yes	unknown

9133G > A	c.8905G > A	V2969M	A	2/380 (0.005)	0/80 (0.000)	yes	unknown

9599A > T	c.9371A > T	N3124I	AT	1/380 (0.003)	0/80 (0.000)	yes	unknown

10462A > G	10234A > G	I3412V	G	1/208 (0.005)	0/80 (0.000)	yes	unknown

**Frameshift changes**							

4091delTAA	c.3863delTAA	del288N	del	2/208 (0.010)	0/80 (0.000)	no	

9512dupl12	c.9284_9295dup12		dup	1/380 (0.003)	0/80 (0.000)	no	

**Intron changes**							

IVS2-7A > T	c.68-7A > T	intron	T	1/208 (0.005)	1/80 (0.013)	yes	unknown

**Synonymous changes**							

1395G > A	c.1167G > A	P389P	A	1/208 (0.005)	0/80 (0.000)	no	

1977G > A	c.1749G > A	L583L	A	1/208 (0.005)	0/80 (0.000)	no	

4296G > A	c.4068G > A	L1356L	A	3/208 (0.014)	0/80 (0.000)	yes	unknown

5013G > A	c.4785G > A	Q1595Q	A	1/208 (0.005)	0/80 (0.000)	no	

5985G > A	c.5757G > A	L1919L	G	0/208 (0.000)	1/80 (0.013)	no	

10338G > A	10110G > A	R3370R	A	2/380 (0.005)	0/80 (0.000)	yes	unknown

10431G > A	10203G > A	T3401T	A	1/208 (0.005)	0/80 (0.000)	no	

Description of nucleotide variations is in accordance with BIC nomenclature* (DNA variants are numerated according to NCBI reference sequence U43746 for mRNA of *BRCA2*.; the first nucleotide of mRNA is numerated as 1) or HGVS nomenclature** (DNA variants are numerated according to NCBI reference sequence NM_000059.3 for *BRCA2*; the first nucleotide of the start codon ATG is numerated as 1).

Unclassified variants were more common in *BRCA2 *(20 different UVs) than in *BRCA1 *(12 different UV) (Tables 4 and 5). Most UVs are missense mutations, yet two UVs in the *BRCA2 *gene cause frame-shift of the open reading frame.

### Controls

The DNA of 40 healthy Slovenian women aged between 50 and 69 years without any personal and familial breast and/or ovarian cancer history was also tested for the presence of all known mutations, UVs and polymorphisms in *BRCA1 *and *BRCA2 *genes in Slovenian population. The results are given in the Tables 2 and 3. No mutations were detected in the control group. All frequent polymorphisms observed in individuals with breast and ovarian cancer family history, with the allele frequency of more than 2%, were detected also in the control group with similar allele frequencies (Table 3).

### Mutation detection rates

The mutation detection rates in different groups and subgroups defined according to the familial cancer history are presented in Tables 6 and 7.

**Table 6:** Incidence of *BRCA1 *or *BRCA2 *mutations among different groups of Slovenian families defined according to family history.

*Group*	*A*	*B*	*C*	*D*	*E*	*MBR*
No. of mutation positive families (%)*						

*BRCA1 *positive	18/77 (23%)	42/100 (42%)	8/75 (11%)	0/21 (0%)	2/24(8%)	0/25 (0%)

*BRCA2 *positive	10/77 (13%)	8/100 (8%)	6/75 (8%)	1/21 (5%)	0/24(0%)	1/25 (4%)

*number of positive families/number of all tested families in the group (the proportion of positive families). *Group A *- families with two or more cases of breast cancer including at least two cases with the disease onset under the age of 50 and with no ovarian cancer. *Group B *- families with one or more cases of breast and at least one ovarian cancer. *Group C *- families with two or more cases of breast cancer including one case diagnosed before the age of 50 and with no ovarian cancer. *Group D *- families with two or more cases of breast cancer all of them diagnosed beyond the age of 50 and with no ovarian cancer. *Group E *- families with a single case of breast cancer diagnosed at the age under 40 years. *Group MBR *- families with a single male breast cancer. Bilateral breast cancers were considered as two separate cases of cancer.

**Table 7:** Incidence of *BRCA1 *or *BRCA2 *mutations among different sub-groups of Slovenian families with breast and ovarian cancer.

*Sub-group*	*BA*	*BC*	*BD*	*1B+1O*	*MoreO+1B*	*Only O*
No. of mutation positive families (%)*						

*BRCA1 *positive	10/19 (53%)	14/21 (67%)	2/7 (29%)	8/32 (25%)	6/7 (86%)	4/14 (29%)

*BRCA2 *positive	1/19 (5%)	2/21 (10%)	2/7 (29%)	3/32 (9%)	0/7 (0%)	1/14 (7%)

*number of positive families/number of all tested families in the group (the proportion of positive families). *Group B *(families with one or more cases of breast and at least one ovarian cancer) was further subdivided into sub-groups: *BA *- families with at least one ovarian cancer and at least two breast cancers diagnosed under the age of 50; *BC *- families with at least one ovarian cancer and at least two breast cancers, one of them diagnosed under the age of 50; *BD - *families with at least one ovarian cancer and at least two breast cancers all of them diagnosed beyond the age of 50; *1B+1O *- families with only one ovarian cancer and one breast cancer in the family or only one affected individual in the family, but having both types of cancer at the same time; *more O+1B *- families with at least two ovarian cancers and only one breast cancer; *only O *- families with at least two ovarian cancers and no breast cancer.

To estimate the influence of number of breast cancer cases in the family on the mutation detection rate among the families with only breast cancer history, we subdivided *Groups A, C *and *D *according to the number of breast cancer cases in the family. The proportions of families with 2, 3 or 4 breast cancers were relatively similar in all three groups (Table 8).

**Table 8:** Incidence of *BRCA1 *or *BRCA2 *mutations among Slovenian families with only breast cancer history (no ovarian cancer) subdivided according to the number of breast cancer cases in the family.

Group	2 breast cancers in family history*	3 breast cancers in family history*	4 breast cancers in family history*	more then 5 breast cancers in family history*
*A*	9/29 (31%)	5/21 (24%)	7/16 (43%)	7/11 (64%)

*C*	5/37 (13%)	3/25 (12%)	4/9 (44%)	2/4 (50%)

*D*	1/10 (10%)	0/6 (0%)	0/5 (0%)	0/0

*number of positive families/number of all tested families in the group (the proportion of positive families). *Group A *- families with two or more cases of breast cancer including at least two of them diagnosed under the age of 50 and with no ovarian cancer. *Group C *- families with two or more cases of breast cancer including one case diagnosed before the age of 50 and with no ovarian cancer. *Group D *- families with two or more cases of breast cancer all of them diagnosed beyond the age of 50 years and with no ovarian cancer.

The frequency at which *BRCA1 *and *BRCA2 *gene mutations were detected in the families belonging to *Group B *was statistically significantly higher when compared to *Groups A, C, D, E *and *MBR *(p-values: 0.034, 0.021, 0.008, 0.006, 0.003, respectively). Also the frequency of mutations seen in *Group A *was statistically significantly different from those observed in *Groups C, D, E *and *MBR*, respectively (p-values < 0.001 in all four cases) (Table 6).

## Discussion

Genetic counseling and testing of individuals from families with an increased risk of breast and/or ovarian cancer in Slovenia has been available at the Institute of Oncology Ljubljana since 1999. From then and until January 2009, we screened 322 families and we detected a highly penetrant *BRCA1 *and *BRCA2 *mutation in 96 of them. In this study, we are summarizing the most significant features of hereditary breast and ovarian cancer in Slovenian families. The overall mutation detection rate in our series was 21.1% for *BRCA1 *and 8.7% for *BRCA2*; for both genes, it was 29.8%.

In general, the mutation detection rates for *BRCA1 *and *BRCA2 *genes in the members of Slovenian families with breast and/or ovarian cancer are comparable with the mutation frequencies in these two genes reported for other countries [15,17,25]. When comparing the study groups, the highest mutation detection rate in Slovenian population was observed in the families with at least one breast and at least one ovarian cancer (*Group B*) - 42% for *BRCA1 *and 8% for *BRCA2 *or 50% for both genes (Table 6). Similar mutation detection rates for families with the same characteristics were reported in other populations - 53% in the German population, 43.7% in the Italian population, 43% in the Spanish population, and 52% in the Dutch population [15,17,23,25].

However, with the additional subdividing of groups in our study, even higher mutation detection rate (of 85%) was observed in the subgroup of families with two or more ovarian cancers and only one breast cancer (*Subgroup - more O+1B*). On the other hand, the proportion of detected mutations in *BRCA1 *and *BRCA2 *genes fell to 36% when we investigated the subgroup of families with at least two ovarian cancers and no breast cancer in their family history (*Subgroup - only O*) (Table 7). Similar findings were perceived by Ramus et al. In their studies, the frequency of *BRCA1 *and *BRCA2 *mutations in the families with site-specific ovarian cancer was lower than in the families with breast and ovarian cancer. The families with three or more ovarian cancers and at least one breast cancer had mutations in *BRCA1 *and *BRCA2 *in 81% of cases (similar to our results) while in the families with three or more ovarian cancers and no breast cancer mutations in *BRCA1 *or *BRCA2 *were detected in only 63% (which is still much higher than in our study) [34,35]. In the Dutch population, the mutation detection rate in the families with at least two ovarian cancers and one or more breast cancers was 82%, whereas in the families with only ovarian cancer history, it was 36%, which is quite similar to our detection rates [17].

This lower mutation detection rate in the families with ovarian cancers only indicates the existence of other (different from *BRCA1/2*) predisposing genes in hereditary ovarian cancers [36]. It is well known that hereditary ovarian cancer can be associated also with Lynch syndrome and with mutations in mismatch repair genes [35].

If we gather all families with at least two breast cancers in their family history regardless of the age at the disease onset and no ovarian cancer, the mutation detection rate in the Slovenian population is 24%. For comparison, the mutation detection rate for *BRCA1 *and *BRCA2 *in similar groups of families in the Dutch, German, Italian and Spanish population is 13%, 24%, 18.5% and 15%, respectively [15,17,23,25]. In the families with two or more breast cancer cases and at least two of them diagnosed under the age of 50 and no ovarian cancer (*Group A*), the mutation detection rate for *BRCA1 *is 23% and for *BRCA2 *is 13% or 36% for both genes in the Slovenian population, 27% in the Dutch population and 37% in the German population [15,17].

In the group of families with no ovarian cancer but at least two or more breast cancer cases with only one of them diagnosed before the age of 50 (*Group C*), the mutation detection rate for *BRCA1 *was 11% and for *BRCA2 *8% or 19% for both genes. These detection rates are slightly higher than the detection rates reported by the German study group (7% and 3% for *BRCA1 *and *BRCA2*, respectively) [15]. In the Dutch population, the mutation detection rate for both *BRCA1 *and *BRCA2 *in this group of families was 15% [17].

When considering our families with at least two breast cancers and all of them diagnosed after age of 50 and no ovarian cancer (*Group D*), the mutation detection rate was 5% (only one mutation discovered in *BRCA2*). In the Dutch population, the reported mutation detection rate in similar group of families was 6% and for the German population 10% [15,17]. Since the mutation detection rate in the families with the same number of breast cancer cases is higher in the group with earlier disease onset (*Group A*) than in the groups with later disease onset (*Groups C and D*), the age at the disease onset seems to influence the *BRCA1/2 *mutation detection rate (Table 8). A rather low mutation detection rate in the group with breast cancers diagnosed at the age over 50 years suggests that other potential predisposing genes confer an increased risk of postmenopausal breast cancer [37].

The families with a single male breast cancer case and no other breast/ovarian cancers among first degree relatives (*Group MBR*) had the mutation detection rate of 4% for *BRCA2 *and 0% for *BRCA1*. Yet, when taken together, all families with male breast cancer in their family history had the mutation detection rate of 11.5% (3 positive families from 26 tested families) [38]. The mutations detected in male breast cancer were c.7806-2A > G and c.3975_3978dupTGCT in *BRCA2 *gene. In comparison, the mutation detection rates in the families with male breast cancer in the German population were 25%, (12 positive cases from 47 tested families) and as high as 42% in Italian (3 positive cases from 7 tested families) and in the Dutch population (5 positive cases from 12 tested families), which is much higher than in our population [15,17,25]. In this place, it must definitely be stressed that, in our study, not all samples of male breast cancer have been fully screened up till now and that only tests for the most frequent mutations in the Slovenian population have been performed. This might be one of the possible reasons for a lower mutation detection rate in this group of families in our population.

Nevertheless, a low detection rate has also been perceived in another group of families with a single case of female breast cancer (*Group E*) where the mutation detection rates for *BRCA1 *and for *BRCA2 *were 8% and 0%, respectively. In the German population, the detection rates in this group are reported to be 8% in *BRCA1 *and 4% in *BRCA2 *[15].

The majority of common mutations in *BRCA1 *(c.181T > G, c.1687C > T, c.5266dupC) observed in the Slovenian population have been frequently described also in other European populations (Table 1), but not the mutation c.844_850dupTCATTAC. This mutation has been previously described only twice in the German population, while it was found in as many as 8 families in the Slovenian population. Another rare mutation in BRCA1 - the mutation c.181T > A has also been previously described only in the Slovenian population [39].

In *BRCA2*, the most frequent mutations were c.7806-2A > G, c.5291C > G and c.3975_3978dupTGCT. The mutation c.7806-2A > G has been already reported by our group [31]. It is a Slovenian founder mutation, reported before also by others [15,40,41]. The mutation c.5291C > G, however, according to BIC database, has not been described in any other populations, other then the Slovenian one.

Different mutations in *BRCA1 *and *BRCA2 *genes have a different penetrance for breast and for ovarian cancer [35]. This has been observed also in our population. The families affected by one mutation actually differ from the families affected by another mutation by means of the proportion of ovarian cancers among all breast and ovarian cancers in the family. These differences can be due to the variations in the biology of the translated protein having a different effect on the normal breast or ovarian tissue. The difference could also be associated with other than *BRCA1 *and *BRCA2 *common moderate/low penetrance genes in the population [35,42,43].

## Conclusion

With the introduction of the genetic councelling and testing into clinical oncology the approach to individuals and families at elevated risk for specific cancer changed radically. It offers namely not only the identification of individuals at risk but also planning of different prevention strategies that may reflect in longer life expectancies of these individuals. In our study, 96 families bearing the *BRCA1 *or *BRCA2 *mutation were identified. The mutation detection rates for *BRCA1 *and *BRCA2 *genes in different groups of families with different family history were similar to the mutation detection rates in other European populations. The most common mutations in the *BRCA1 *gene in the families with an increased risk of breast and/or ovarian cancer in Slovenia are c.181T > G, c.1687C > T, c.5266dupC, c.844_850dupTCATTAC and c.181T > A. The most frequent mutations in *BRCA2 *are c.7806-2A > G, c.5291C > G and c.3975_3978dupTGCT. The cumulative share of these frequent mutations in *BRCA1 *and *BRCA2 *genes in Slovenian families affected with mutations is 68.7%. In the Slovenian population, the mutation detection rate seems to be influenced by the presence of ovarian cancer history, the age at the diagnosis of breast cancers in the family and the number of breast cancer cases in the family. As well, the mutation detection rate might further be influenced in the future - it might namely increase when the character of some of the unclassified variants will be clarified as pathogenic.

## Competing interests

The authors declare that they have no competing interest.

## Authors' contributions

VS, MK, JŽ, MH, ET, JG and SN designed the study, collected and analyzed the data and wrote the paper.

## Pre-publication history

The pre-publication history for this paper can be accessed here:

http://www.biomedcentral.com/1471-2350/12/9/prepub
